# Compact Modeling of Advanced Gate-All-Around Nanosheet FETs Using Artificial Neural Network

**DOI:** 10.3390/mi15020218

**Published:** 2024-01-31

**Authors:** Yage Zhao, Zhongshan Xu, Huawei Tang, Yusi Zhao, Peishun Tang, Rongzheng Ding, Xiaona Zhu, David Wei Zhang, Shaofeng Yu

**Affiliations:** 1 School of Microelectronics, Fudan University, Shanghai 200433, China; 20112020044@fudan.edu.cn (Y.Z.); 21112020147@m.fudan.edu.cn (Z.X.); 23112020147@m.fudan.edu.cn (H.T.); 22212020147@m.fudan.edu.cn (P.T.); xnzhu@fudan.edu.cn (X.Z.); dwzhang@fudan.edu.cn (D.W.Z.); 2 National Integrated Circuit Innovation Center, Shanghai 201203, China

**Keywords:** gate-all-around (GAA) Nanosheet FETs (NSFETs), compact model, artificial neural network (ANN), TCAD simulation

## Abstract

As the architecture of logic devices is evolving towards gate-all-around (GAA) structure, research efforts on advanced transistors are increasingly desired. In order to rapidly perform accurate compact modeling for these ultra-scaled transistors with the capability to cover dimensional variations, neural networks are considered. In this paper, a compact model generation methodology based on artificial neural network (ANN) is developed for GAA nanosheet FETs (NSFETs) at advanced technology nodes. The DC and AC characteristics of GAA NSFETs with various physical gate lengths (*L*_g_), nanosheet widths (*W*_sh_) and thicknesses (*T*_sh_), as well as different gate voltages (*V*_gs_) and drain voltages (*V*_ds_) are obtained through TCAD simulations. Subsequently, a high-precision ANN model architecture is evaluated. A systematical study on the impacts of ANN size, activation function, learning rate, and epoch (the times of complete pass through the entire training dataset) on the accuracy of ANN models is conducted, and a shallow neural network configuration for generating optimal ANN models is proposed. The results clearly show that the optimized ANN model can reproduce the DC and AC characteristics of NSFETs very accurately with a fitting error (MSE) of 0.01.

## 1. Introduction

In response to market demands, the transistor dimensions have been scaled down proportionally according to Moore’s Law. As an alternative to planar metal-oxide-semiconductor field-effect transistors (MOSFETs), fin field-effect transistors (FinFETs), which utilize a three-dimensional architecture with the gate wrapping around vertical fins on top and sides, have been developed and commercialized in 22 nm CMOS technology [1,2,3]. During the past decades, FinFET technology has been successfully applied to 5 nm and even 3 nm technology nodes through higher aspect ratio and layout optimization [4,5,6,7,8]. However, the scaling of FinFETs has also encountered fabrication- and performance-related obstacles due to fundamental physical limitations and difficulties in developing the required process. The performance improvement is constrained by the severe short channel effects (SCEs), while it is difficult to populate multiple fins in a limited space as CGP is further reduced. As the most feasible solution to extend Moore’s Law and Dennard’s Law, gate-all-around (GAA) nanosheet FETs (NSFETs) are poised to become a mainstream device architecture for 2 nm node and beyond [9,10,11,12]. Compared to traditional FinFETs or planar MOSFETs, GAA NSFETs offer superior electrostatic control, higher driving capability, lower leakage current, and more effective footprint [10]. This is because they not only have the gates surrounding the channel, but also have wider effective widths in the same footprint. Nevertheless, this advancement imposes a challenge to semiconductor device models.

Semiconductor device models are regarded as a bridge between foundry, EDA vendor, and design house, as well as a key-enabler for accurate integrated circuit (IC) simulations. The conventional semiconductor device models include macro models [13,14], compact models [15,16,17,18], and look-up table (LUT) models [19,20]. In particular, compact models are the mainstream ones and are composed of physics-based equations, which have been developed for decades. The first industry standard compact model is BSIM (Berkeley short-channel insulated-gate field-effect transistor model), whose genesis can be traced to the 1980s [21], and several versions have been developed and remain in use today [16,22,23]. Generally, analytical equations are used to describe device *I*–*V* and *C*–*V* characteristics in the subthreshold, linear, and saturation regions in a unified way. The accuracy of the compact models is crucial for efficient analysis and design of ICs. However, for advanced transistors, the underlying physics becomes much more complicated, making the models more difficult to fit. In addition, the actual electrical properties of miniaturized transistors are case sensitive due to dimension variations. Since developing suitable analytical compact models is complex and often takes several years, it requires novel modeling methodology to circumvent the high costs of time and labor.

The need for a new technique brings the artificial neural network (ANN) method to the attention of researchers, which has been attempted for planar MOSFETs modeling since the early 1990s and showed good precision [24]. ANNs represent a class of machine learning models inspired by the neuromorphic architecture, and use a set of multilayered perceptrons/neurons, also known as feed-forward neural networks, consisting of an input layer, multiple hidden layers, and an output layer [25,26]. Because of the robust learning capability, they have once been a powerful tool used in the computer science to deal with machine learning issues. The primary objective of ANNs is to learn complex mappings between inputs and outputs by adjusting the weights and biases of interconnected neurons, in a nutshell, is to achieve a good means of solving data fitting problems. This learning process involves the application of mathematical principles, particularly the chain rule in calculus, to update the network parameters and minimize the error between predicted and actual outcomes. In other words, with a reasonable network configuration, ANNs can fit arbitrary nonlinear functions and hence can also be developed as black-box models to address nonlinear systems or more sophisticated internal expressions, such as the compact modeling of semiconductor devices in advanced nodes mentioned earlier. Although the ANN models seemed to be a simple black box, there are many parameters within the neural network that have an impact on the accuracy of models, which will further affect the subsequent circuit simulations. Thereby, an in-depth study of ANN-based compact modeling methodology is necessary for the development and application of GAA devices and even complementary FET (CFET) devices, which are more sophisticated architectures with n-FET folded onto p-FET, in advanced technologies [27,28]. Actually, there are some interesting and meaningful studies on ANN-based device modeling that have been published in recent years [29,30,31,32]. However, most of the literature in this field have only superficially studied ANN modeling, focusing instead on its implementation in subsequent circuits or on the unique electrical properties under investigation, and lacking an in-depth understanding and full exploration of the ANNs used for modeling.

In this work, we conduct a comprehensive evaluation of the compact modeling of advanced GAA NSFETs based on ANN, with the datasets from finely calibrated TCAD simulations. Referring to [10] and IRDS 2022 [33], an N-channel GAA NSFET was built as the nominal transistor for the modeling study. The applied voltages on terminals and 3-D nanosheet dimensions were set as input parameters and varied to obtain datasets, some of which were used for training data feeding into the ANN and the others were used for testing data for the final test. Appropriate data preprocessing and neural network configurations, as well as *L*2 regularization were adopted to improve model accuracy. Without considering the physical characteristics of real transistors, high fitting accuracy can be achieved by using transistor data for model training. The DC and AC characteristics are well mapped with the five input variants, including applied voltages and geometrical dimensions.

## 2. Device Structure, TCAD Simulation Calibration, and Dataset Generation

### 2.1. Device Structure

**Figure 1:**
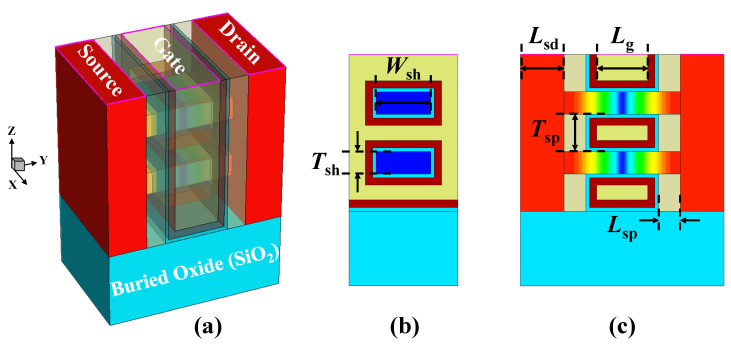
An illustration of nominal gate-all-around nanosheet FET (GAA NSFET) and details of device structure: (**a**) Entire 3-D schematic; (**b**) X–Z cut plane and (**c**) Y–Z cut plane of the nominal device structure.

The Sentaurus Technology Computer-Aided Design (TCAD) [34] tool is exploited to construct the GAA NSFET devices and generate physical electric characteristic data for subsequent studies. Figure 1a–c shows the 3-D schematic of nominal GAA NSFET structure and 2-D cross-sectional along and across the channel views, respectively. Detailed parameters of a nominal highly scaled device at 2 nm technology node are specifically listed in Table 1 following IRDS 2022 [33], where the physical gate length (*L*_g_) of 14 nm, nanosheet width (*W*_sh_) of 15 nm, nanosheet thickness (*T*_sh_) of 6 nm, the spacer length (*L*_sp_) of 6 nm, and the sheet-to-sheet spacing (*T*_sp_) of 10 nm are adopted. For n-type MOS, the in-situ uniform doping profiles for channels and source/drain regions were performed with 1 × 10^10^ cm^−3^ of boron doping concentration and 5 × 10^20^ cm^−3^ of arsenic doping concentration, respectively. As for the high-k/metal gate (HKMG) stack, the equivalent oxide thickness (EOT) is 1.35 nm, which consists of HfO_2_ of 2 nm and interfacial oxide SiO_2_ of 1 nm. The work-function metal used in the gate stack is TiN and the effective work-function (*WF*) is set to 4.4 eV. Note that for high-performance devices, the geometric parameters are the same as above except for the nanosheet width being wider.

### 2.2. TCAD Simulation Calibration

Since nanoscale devices typically exhibit size-dependent behavior, the corresponding physical model parameters built-in in the TCAD simulator may not be accurate enough with the scale shrinking, which affects the validity of the device characteristics resulted from TCAD simulations. Therefore, in order to ensure the accuracy of the subsequent simulations to generate more physically accurate datasets for the subsequent ANN model, it is essential to calibrate the simulator against experimental data to lay a solid ground for the ANN modeling work. In this calibration work, both DC and AC characteristics were covered, comprehensively demonstrating the exactitude of the simulation platform.

**Figure 2:**
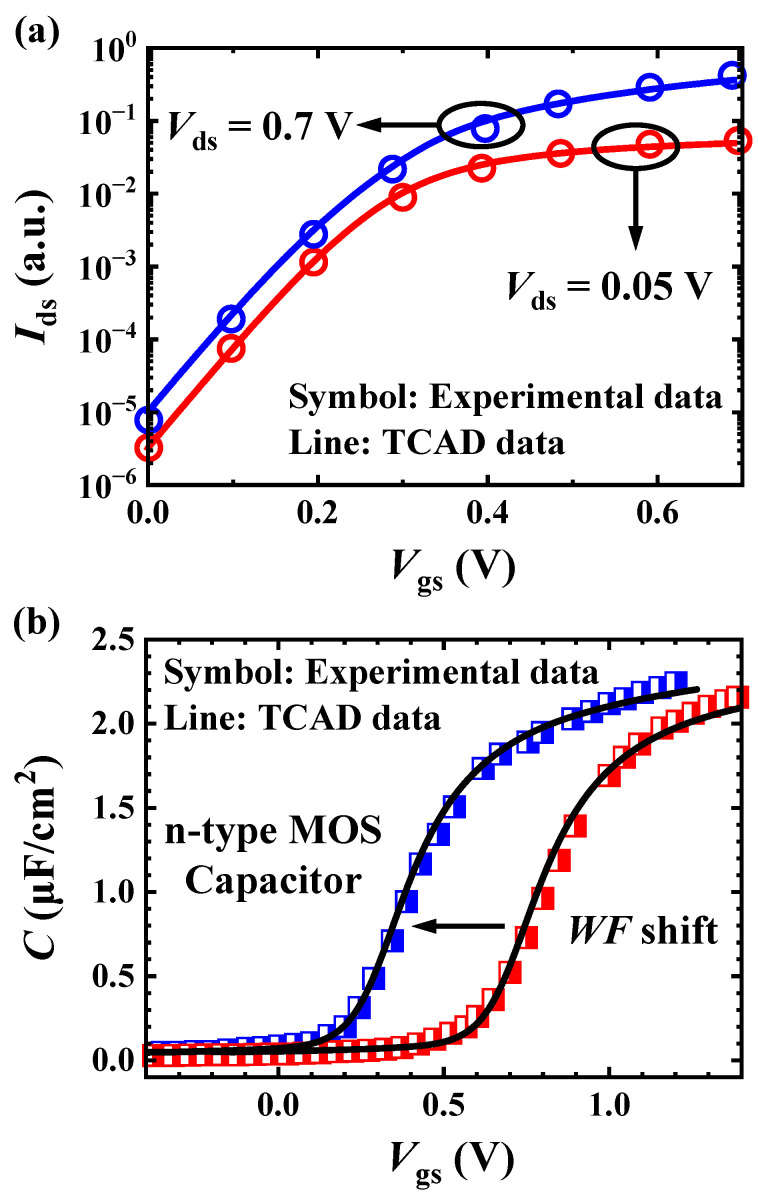
TCAD simulation calibrations against the experimental data under the same simulation environment. (**a**) Calibrated *I*_ds_–*V*_gs_ characteristics of n-type NSFET versus experiment data from Ref. [10], and (**b**) *C*–*V* characteristics of n-type MOS capacitor versus experimental data from Ref. [35].

Besides the nominal GAA NSFET structure illustrated in the previous section for DC calibration, an n-type MOS capacitor was generated according to the device description for AC calibration [35]. TCAD calibrations against experimental data of Refs. [10,35] andwere performed in the framework of drift-diffusion (DD) transport model with quantum correction in electrostatics. The results are shown in Figure 2a,b, where the calibrated simulator closely matches the experimental *I*_ds_–*V*_gs_ and *C*–*V* characteristics after adjustments of the relevant model parameters. The physical models used include the Philip unified mobility, thin-layer mobility and high-field saturation models, as well as Shockley–Read–Hall (SRH) recombination, Auger recombination and band-to-band tunneling models in the drift-diffusion (DD) framework. Physically more correct, Fermi-Dirac statistics are used for high doping concentrations. Furthermore, the density-gradient and kinetic velocity models are considered to account for quantum confinement and ballistic effects.

### 2.3. Dataset Generation

Based on the previously calibrated simulation environment, a number of GAA NSFETs were designed by altering the nanosheet dimensions (*L*_g_, *W*_sh_, and *T*_sh_) of the nominal device. And the ranges of dimensional variants were designed to cover the specifications of IRDS roadmap organized for 3 nm to 1 nm nodes [33]. Here, *L*_g_ ranges from 10 to 20 nm, *W*_sh_ ranges from 15 to 30 nm, while *T*_sh_ has a smaller movable range between 4–7 nm. Then, the DC and AC characteristics were extracted to create dataset when *V*_ds_ and *V*_gs_ were set at 0–0.7 V. For the C-V model, the AC characteristics were obtained with a frequency of 10^6^ Hz. In the practical simulation experiments, we can flexibly control the number of points taken in the electric characteristic curves. Considering that too much data generated by TCAD are very likely to cause overfitting and waste of computing resource, we finally randomly selected 4000 sets of data to form the dataset used for the subsequent study.

## 3. Development and Optimization of ANN Model

### 3.1. Development of ANN Model

**Figure 3:**
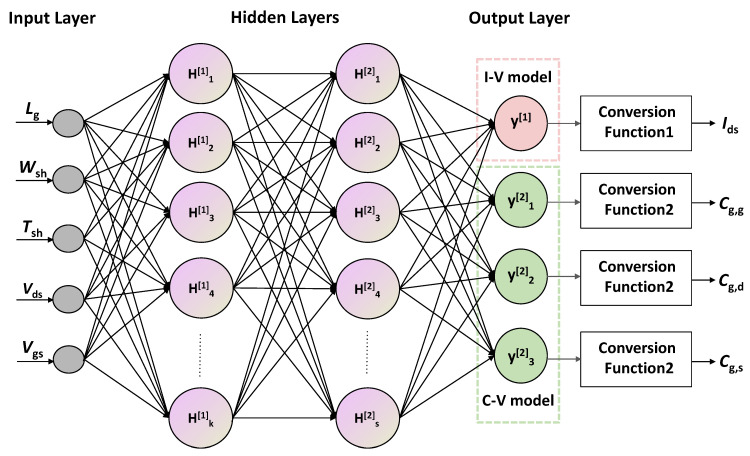
The regression neural network topology framework.

Figure 3 shows the proposed schematic diagram of developing a regression ANN model, which is executed in the following steps: (1) accepting the input data, (2) fine-tuning the input and output parameters while training the model, (3) testing and (4) evaluating the trained model using the testing data. A complete five parameters are used as input variants, including gate-to-source voltage (*V*_gs_), drain-to-source voltage (*V*_ds_), physical gate length (*L*_g_), nanosheet width (*W*_sh_), and nanosheet thickness (*T*_sh_). The training/testing data, which comprising DC and AC characteristics for various input parameters, is obtained from physical TCAD simulations. The hidden layers consist of two layers, with *k* (k=10) and *s* (s=5) neurons respectively. The number of neurons in the output layer is *p* (p=4), one is used for the *I*–*V* model, and the other three are used for the *C*–*V* model. Besides, we define the conversion function for mapping the output values of the ANN model to the real current *I*_ds_ and the capacitance *C*_g,g_, *C*_g,d_ and *C*_g,s_. The training of the ANN model is realized using python with the assistance of the PyTorch package.

In our ANN model, each hidden layer consists of multiple neurons, and the connections between neurons are characterized by weights *w* and biases *b*. The mathematical foundation of ANNs relies on the activation function, often denoted as *f*, which introduces non-linearity into the model. The training of the network includes two steps: the forward process and the backward process. The forward process of the network involves calculating the weighted sum of inputs, applying the activation function, and passing the result to the next layer. This process is repeated layer by layer until the final output is obtained. The mathematical representation of the forward process can be expressed as follows:

(1) $$net_{j}^{\left(k\right)}=\sum_{i=1}^{n^{\left(k-1\right)}}w_{j,i}^{\left(k\right)}*y_{i}^{\left(k-1\right)}+b_{j}^{\left(k\right)}$$

(2) $$y_{j}^{\left(k\right)}=f\left(net_{j}^{\left(k\right)}\right)$$

Here, $net_{j}^{\left(k\right)}$ represents the weighted sum of inputs for neuron *j* in layer *k*, $w_{j,i}^{\left(k\right)}$ denotes the weight connecting neuron *i* in layer k−1 to neuron *j* in layer *k*, $y_{i}^{\left(k-1\right)}$ is the output of neuron *i* in layer k−1, $b_{j}^{\left(k\right)}$ is the bias for neuron *j* in layer *k*, and $f(net_{j}^{\left(k\right)})$ is the activation function. Especially, the hyperbolic tangent function tanh(x) was used as the activation function [36,37]. The output of tanh(x) lies within the range of $\left[-1,1\right]$, which, compared to the $\left[0,1\right]$ range of the sigmoid function, makes tanh(x) advantageous in zero-centering. This helps mitigate the exploding gradient problem during gradient descent.

The training process involves minimizing a predefined loss function, typically the mean squared error (MSE), which measures the discrepancy between the predicted and actual outputs. MSE is calculated by taking the average of the squared differences between predicted and actual values, which is a simple and easily differentiable form. This simplicity facilitates the updating of weights in optimization algorithms like gradient descent. In addition, as MSE involves squaring the errors, it is less sensitive to outliers (samples with significantly different actual values). This means that individual outliers do not have a disproportionately large impact on the overall loss function, enhancing the robustness of the model.

The backward process, also known as backpropagation, is a crucial step in updating the network parameters. The gradients are propagated backward through the network, and the weights and biases are adjusted using optimization algorithms such as stochastic gradient descent (SGD) [38]. The chain rule is applied iteratively to compute the gradients of the loss (*L*) with respect to the network parameters:

(3) $$\frac{\partial L}{\partial w_{j,i}^{\left(k\right)}}=\frac{\partial L}{\partial net_{j}^{\left(k\right)}}*\frac{\partial net_{j}^{\left(k\right)}}{\partial w_{j,i}^{\left(k\right)}}$$

(4) $$\frac{\partial L}{\partial b_{j}^{\left(k\right)}}=\frac{\partial L}{\partial net_{j}^{\left(k\right)}}*\frac{\partial net_{j}^{\left(k\right)}}{\partial b_{j}^{\left(k\right)}}$$

These gradients guide the parameters update during the training process, gradually optimizing the network to improve its predictive capabilities. The iterative nature of backpropagation allows the network to learn complex patterns and relationships within the data.

Thus, a four-layered regression ANN involves intricate mathematical formulations, including the forward pass equations for computing neuron activations and the backward pass equations for updating weights and biases during training. The application of the chain rule in calculus is fundamental to these computations, enabling the network to learn and adapt to complex patterns in the data.

### 3.2. Optimization of ANN Model

Before the training process, we noticed that the orders of magnitude of the outputs are too small, 10^−13^∼10^−3^ for the *I*–*V* model and 10^−18^∼10^−17^ for the *C*–*V* model, which are not favorable for data fitting. So, we preprocessed the outputs (*I*_ds_, *C*_g,g_, *C*_g,d_, and *C*_g,s_) in order to achieve the accurate fitting through a linear preprocessing method. Here, we multiplied the output currents and capacitances by factors of 1 × 10^6^ and 1 × 10^18^, respectively, thereby converting the units from A and F to μA and aF.

Since then, the processed dataset was utilized for training, but another problem was identified, namely, the ANN model had overfitting, which means it performs well during training but fails to generalize effectively to the test samples. In other words, our model has a significant gap between the model’s performance during training and its performance when making predictions on new data. The network excels in fitting the training data but struggles to make accurate predictions on unseen examples.

Overfitting often leads to excessively complex neural network models. These models tend to capture noise and outliers in the training data, making them less suitable for generalization. Moreover, the loss function used during training may not accurately reflect the network’s performance on new data. The model might minimize the training loss, giving a false sense of success, while failing to minimize the loss on validation or test data. Let $L_{\mathrm{train}}$ denotes the training loss, $L_{\mathrm{val}}$ the validation loss, and $L_{\mathrm{test}}$ the test loss. Overfitting occurs when $L_{\mathrm{train}}$ is significantly smaller than both $L_{\mathrm{val}}$ and $L_{\mathrm{test}}$.

(5) $$L_{\mathrm{train}}\ll L_{\mathrm{val}},L_{\mathrm{test}}$$

To address this issue, we adopt L2 regularization (also known as weight decay) [39], which is a widely adopted technique to address overfitting by adding a penalty term to the loss function. The regularized loss function is given by:

(6) $$L=\frac{1}{2}{∥Xw-y∥}^{2}+\lambda {∥w∥}^{2}$$

Here, *X* is the input matrix, *w* is the weight vector, *y* is the target vector, and λ is the regularization parameter that controls the strength of the regularization. The first term $\frac{1}{2}{∥Xw-y∥}^{2}$ represents the MSE (described in Section 3.1), aiming to minimize the difference between the predicted and actual values. The second term $\lambda {∥w∥}^{2}$ is the L2 regularization term. It penalizes large weights by adding the squared magnitude of the weight vector. The regularization parameter λ controls the trade-off between fitting the training data and preventing overfitting.

From the viewpoint of convex optimization, the introduction of the L2 regularization term transforms the optimization problem into a constrained optimization problem. The regularization term induces a constraint on the magnitude of the weight vector, effectively defining a hypersphere in the weight space. This transformation has a smoothing effect on the optimization landscape, making it more convex. The regularization term adds a regularization force that discourages the weights from reaching extreme values, leading to a more stable and generalizable model.

In summary, L2 regularization mitigates overfitting by penalizing large weights in a linear regression model. The mathematical formulation introduces a balance between fitting the training data and controlling the complexity of the model. From a convex optimization perspective, the regularization term induces a constraint that shapes a more well-behaved optimization landscape.

## 4. Results and Discussion

**Figure 4:**
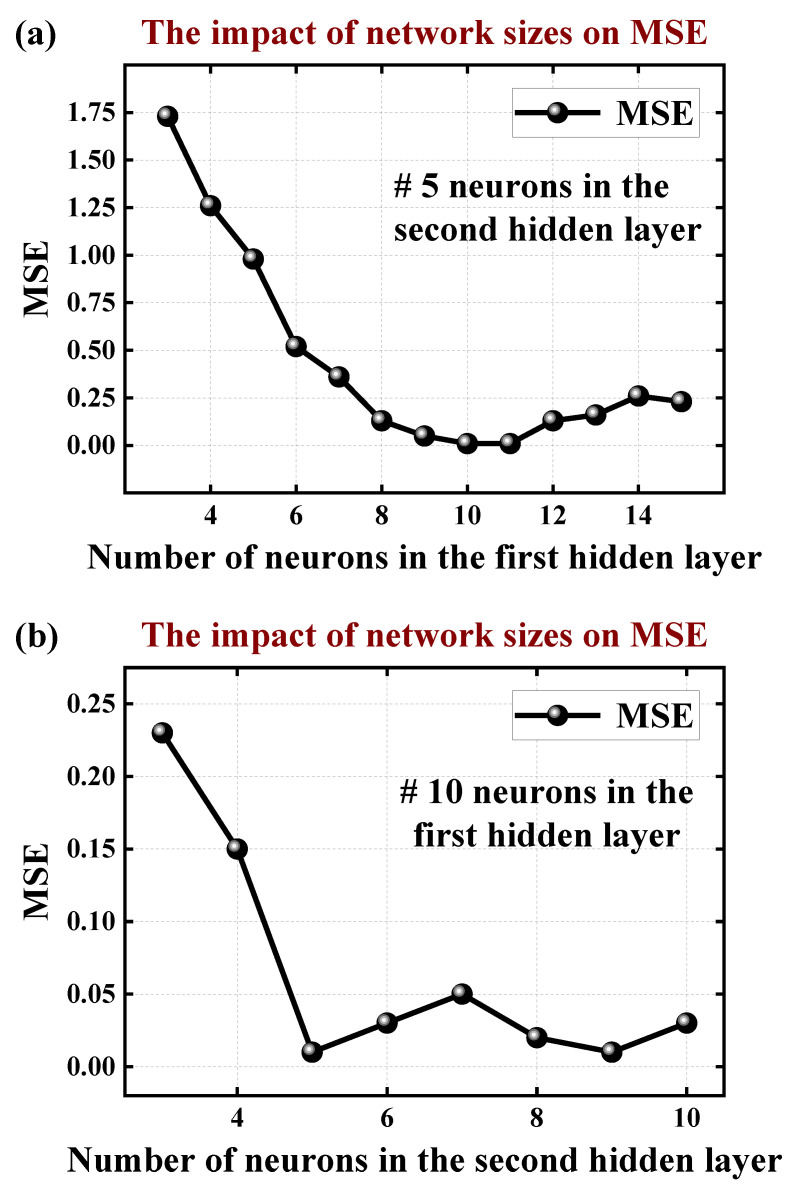
The MSE for all the test samples with different numbers of neurons in the first hidden layer (**a**) and second hidden layer (**b**).

A total of 4000 samples for ANN training and testing are obtained from *I*_ds_–*V*_gs_ and *C*–*V* data generated by previous TCAD simulations. We randomly split these samples into a training set (a total of 3200 samples) and a testing set (a total of 800 samples) in a 4:1 ratio. Theoretically, as the number of hidden layers and neurons increases, the ANN model becomes more capable of extracting the non-linear mapping relationship between input and output. However, in practice, too many hidden layers or number of neurons can also bring about overfitting problems. And in most cases, the fitting accuracy is determined synergistically by both the number of hidden layers and neurons. For most fitting cases with limited input and output variants, a shallow neural network is sufficient, which is easier to be trained and converges to the optimal solution faster, with a more favorable computational and memory footprint. Thus, to obtain an optimal network, we studied the impact of network sizes on the errors (MSE) for shallow neural networks with two hidden layers. As shown in Figure 4, we find that the MSE of the testing set tends to decrease and then increase as the number of neurons increases. The minimum MSE is 0.01 with ten neurons in the first hidden layer and five neurons in the second hidden layer.

**Figure 5:**
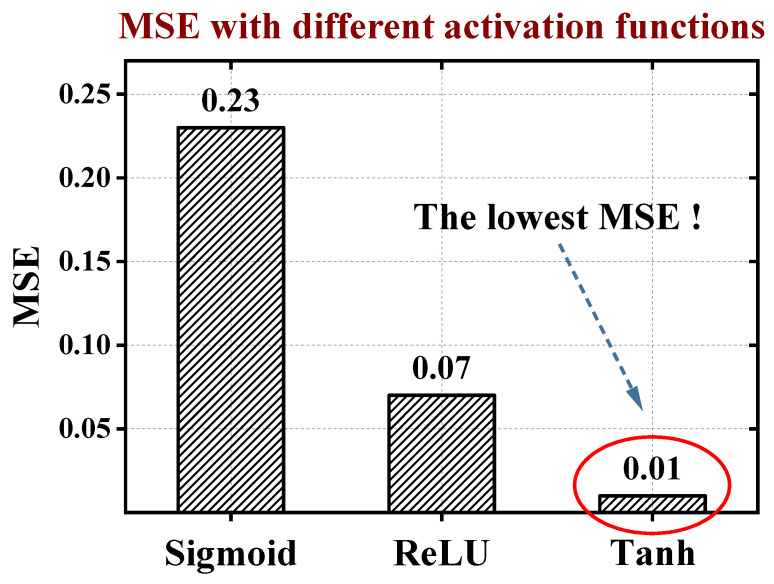
The MSE with different activation functions of the neurons. Popular activation functions: sigmoid, relu, and tanh.

Besides, we investigated the impact of different types of activation functions of the neurons on MSE for the test dataset, as depicted in Figure 5. Using the hyperbolic tangent function tanh(x) has the lowest MSE. Through our analysis, the tanh(x) function is zero-centered, meaning its mean is zero. This is beneficial for optimization algorithms such as gradient descent, as it helps prevent the gradient updates from consistently favoring a particular direction, thus improving the convergence speed of the model. The derivative of the tanh(x) function is non-zero in most regions, aiding in the propagation of gradients during backpropagation. Unlike the sigmoid function, the gradient of tanh(x) does not approach zero in regions of large or small inputs, reducing the risk of the vanishing gradient problem.

**Figure 6:**
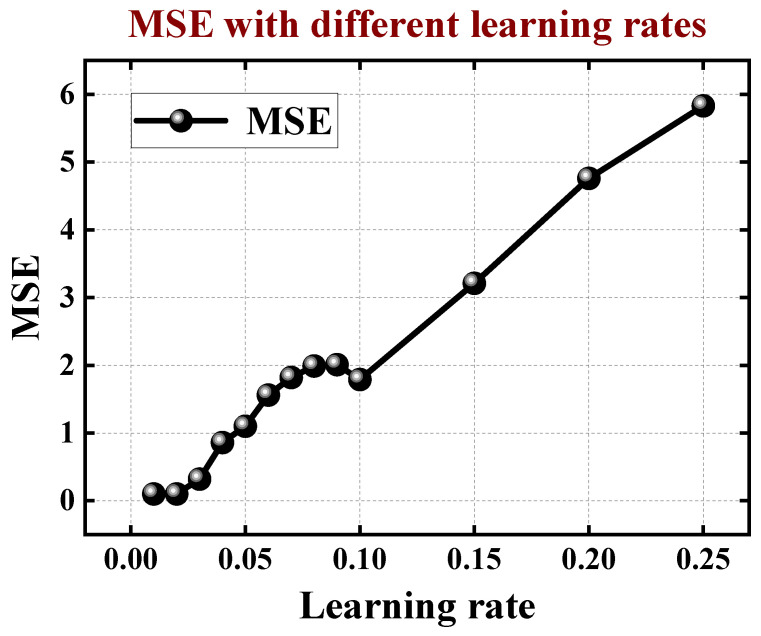
The MSE with different learning rates.

Moreover, we have studied the impact of the learning rate on MSE. In our ANN model, the learning rate is a crucial hyper parameter in training neural networks. It controls the magnitude of updates applied to the weights during the training process. A higher learning rate means larger updates, leading to faster convergence but with the risk of overshooting the optimal weights. Conversely, a lower learning rate allows for smaller weight updates, potentially resulting in slower convergence but increased precision in finding the global minimum. Through our experiments, we think learning rate of 0.02 is the best solution for the ANN model as shown in Figure 6.

**Figure 7:**
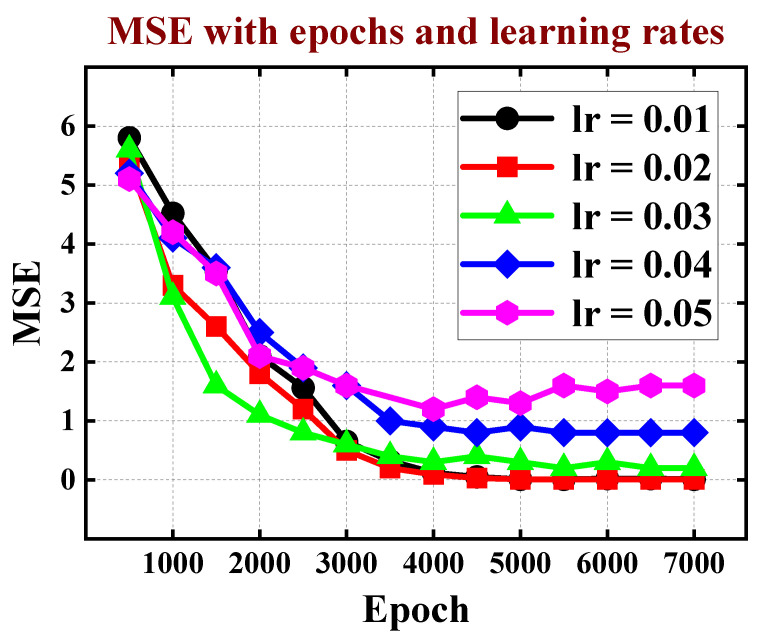
The MSE decline process with different learning rates as epoch increases.

Epochs represent the number of times the entire dataset is fed forward and backward through the neural network during the training process. The choice of the number of epochs plays a pivotal role in determining how well the model generalizes to unseen data. Too few epochs may result in under fitting, where the model fails to capture the underlying patterns in the data. On the other hand, an excessive number of epochs may lead to overfitting, causing the model to memorize the training data but perform poorly on new, unseen data. Moreover, the relationship between epoch and learning rate is interdependent. A higher learning rate may require fewer epochs to converge, as each iteration leads to more substantial weight updates. Conversely, a lower learning rate might necessitate a higher number of epochs to allow the model to converge gradually. Finally, as shown in Figure 7, we choose the lowest MSE (0.01) scheme with the epoch of 5000 and the learning rate of 0.02.

**Figure 8:**
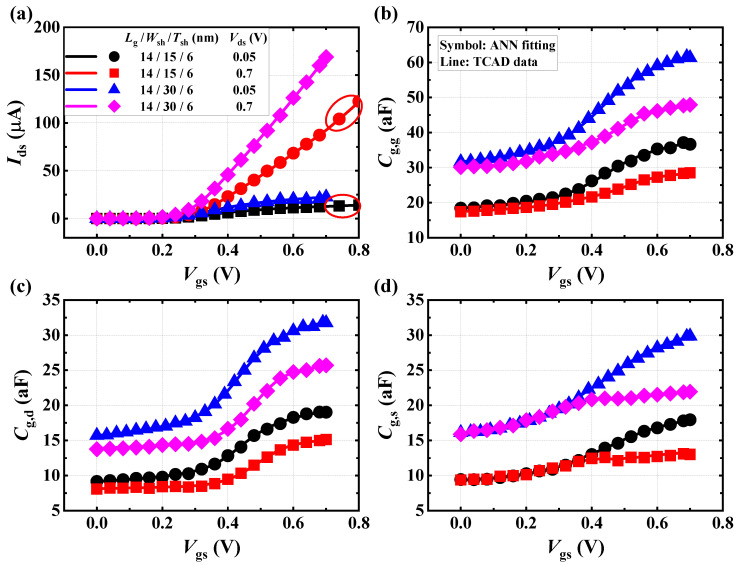
Example ANN model fitting results of the simulated DC and AC characteristics for high-density and high-performance GAA NSFETs at 2 nm technology node. (**a**) *I*_ds_–*V*_gs_. (**b**) *C*_g,g_–*V*_gs_. (**c**) *C*_g,d_–*V*_gs_. (**d**) *C*_g,s_–*V*_gs_.

Here, we summarize the primary parameters of the proposed ANN model, as shown in Table 2. Based on the experiments and analysis, we can utilize the ANN model to predict the current and capacitance output based on input data (*L*_g_, *W*_sh_, *T*_sh_, *V*_gs_ and *V*_ds_) with MSE of 0.01. As shown in Figure 8, the example results of both the DC and AC characteristics of ANN model are fitted against the TCAD data of high-density and high-performance GAA NSFETs at 2 nm technology node. It can be seen that the output *I*–*V* and *C*–*V* performances generated by ANN fit well with the TCAD results. In addition, we predicted the *I*–*V* performance of the nominal NSFET device at high gate bias (*V*_gs_ = 0.7–0.8 V) to examine the model scalability. The extrapolation behavior also fits well to simulation results. The optimistic results reveal that the proposed network is capable of handling the electrical characterization of advanced GAA NSFETs with great accuracy.

## 5. Conclusions

In summary, the ANN-based compact modeling methodology has been thoroughly investigated for advanced GAA NSFETs. Here, the impacts of ANN size, activation function, learning rate, and epoch on the accuracy of ANN models were systematically evaluated. Based on the precisely calibrated simulation environment, various GAA NSFET devices were constructed by varying the nanosheet dimensions, and their DC as well as AC characteristics were extracted. The generated dataset contains five input variants (*V*_gs_, *V*_ds_, *L*_g_, *W*_sh_, and *T*_sh_) and four output quantities (*I*_ds_, *C*_g,g_, *C*_g,d_, and *C*_g,s_). Before the training process, the output data were preprocessed to circumvent unnecessary fitting mistakes using a linear preprocessing method. By adopting the *L*2 regularization, the overfitting issue was perfectly resolved with the addition of a penalty term to the loss function. The optimized ANN model fully demonstrates its superior fitting properties under various conditions with a low fitting MSE error of 0.01. Furthermore, the scalability was also validated. This work contributes to the development of ANN-based compact models, holding great promise for adoption in advanced fast turn-around design and technology co-optimization (DTCO) as well as large-scale product-design-oriented circuit simulations.

## Figures and Tables

**Table 1:** Detailed parameters of nominal device at 2 nm technology node [33].

Parameters	Value
Physical gate length (*L*_g_)	14 nm
Source/drain length (*L*_sd_)	12 nm
Spacer length (*L*_sp_)	6 nm
Nanosheet width (*W*_sh_)	15 nm
Nanosheet thickness (*T*_sh_)	6 nm
Sheet-to-sheet spacing (*T*_sp_)	10 nm
Equivalent oxide thickness (*EOT*)	1.35 nm
Source/drain doping concentration (*N*_sd_)	5 × 10^20^ cm^−3^
Channel doping concentration (*N*_ch_)	1 × 10^10^ cm^−3^
Metal gate work-function (*WF*)	4.4 eV

**Table 2:** The primary parameters of the ANN model.

Parameters	Features
Network size	5-10-5-4
Activation function	Hyperbolic tangent function
Learning rate	0.02
Epoch	5000
#Training samples	3200
#Test samples	800
Task	Regression
MSE	0.01
Regularization	*L*2 Regularization

## Data Availability

The data and code are available from the corresponding authors upon reasonable request.
